# How to Stir Up Trouble…while Riding a Rollercoaster

**DOI:** 10.1371/journal.pmed.1001520

**Published:** 2013-09-24

**Authors:** Virginia Barbour

**Affiliations:** PLOS Medicine, Cambridge, United Kingdom

## Abstract

*PLOS Medicine*'s Chief Editor looks back at some highlights in the way the journal has sought to constructively disrupt medical publishing over its history.

*Please see later in the article for the Editors' Summary*

The phrase that best sums up my time at *PLOS Medicine* is from a tremendously skilled lawyer who has held our hands through some difficult times:

“I'm always happy to help provide guidance on how to stir up trouble without getting into too much yourself.”

Is that too disruptive? On the contrary—the history of PLOS is of an innovative organization started by people with a vision of changing publishing, who employed staff who shared that vision, who themselves also understood the need for disruption as well as the pragmatic realities of publishing (in my case medical publishing) to make that vision a reality. Many times over the following years we have debated our purpose and strategy and concluded with certainty that the best way to innovate in publishing was to challenge assumptions head on, to take the debate to the field.

We have published many excellent research and magazine articles since the journal began, but some of the articles I am most proud of exemplify that disruption and are not the obvious fare of medical journals. Among these are papers on: why lethal injection is not a humane method of killing, as it probably asphyxiates prisoners—which led to lethal injection being ruled unconstitutional in the state of Tennessee [1]; a method of measuring how bad a war is—which led to a change in NATO procedures in Southern Afghanistan [2]; and a forensic dissection of articles we helped bring to light [3], though a legal intervention, on how companies manipulate doctors through ghostwritten journal articles [4]. See Box 1 for more of PLOS Medicine's disruptive content.

**Box 1. Disruptive Content on *PLOS Medicine***

**Lethal Injection**

- Zimmers TA, et al. (2007) Lethal Injection for Execution: Chemical Asphyxiation? PLoS Med 4(4): e156. doi:10.1371/journal.pmed.0040156.

**Measuring the Impact of War**

- Hicks MH-R, et al. (2008) The Dirty War Index: A Public Health and Human Rights Tool for Examining and Monitoring Armed Conflict Outcomes. PLoS Med 5(12): e243. doi:10.1371/journal.pmed.0050243.

**Ghostwriting**

- Fugh-Berman AJ (2010) The Haunting of Medical Journals: How Ghostwriting Sold “HRT”. PLoS Med 7(9): e1000335. doi:10.1371/journal.pmed.1000335.
- The Wyeth Ghostwriting Archive: http://www.plosmedicine.org/static/ghostwriting

**Big Food**

- Stuckler D, et al. (2012) Big Food, Food Systems, and Global Health. PLoS Med 9(6): e1001242. doi:10.1371/journal.pmed.1001242
- The *PLOS Medicine* Series on Big Food: http://www.ploscollections.org/article/browse/issue/info%3Adoi%2F10.1371%2Fissue.pcol.v07.i17

**Big Pharma**

- Kesselheim AS, Mello MM, Studdert DM (2011) Strategies and Practices in Off-Label Marketing of Pharmaceuticals: A Retrospective Analysis of Whistleblower Complaints. PLoS Med 8(4): e1000431. doi:10.1371/journal.pmed.1000431

**Big Alcohol**

- McCambridge J, Hawkins B, Holden C (2013) Industry Use of Evidence to Influence Alcohol Policy: A Case Study of Submissions to the 2008 Scottish Government Consultation. PLoS Med 10(4): e1001431. doi:10.1371/journal.pmed.1001431

**Big Tobacco**

- Weishaar H, Collin J, Smith K, Grüning T, Mandal S, et al. (2012) Global Health Governance and the Commercial Sector: A Documentary Analysis of Tobacco Company Strategies to Influence the WHO Framework Convention on Tobacco Control. PLoS Med 9(6): e1001249. doi:10.1371/journal.pmed.1001249

I'm now moving to Australia and as a result felt that the time was right to step down as Chief Editor. However, I am remaining with PLOS, in the other post I also hold as Medicine Editorial Director. In this position, I will have oversight of the medical journals (*PLOS Medicine*, *PLOS Neglected Tropical Diseases*, and *PLOS Pathogens*) but no day-to-day input into content. I very much look forward to engaging with medical publishing in a new part of the world and will continue to blog, tweet, and agitate from there. I'm delighted to say that my successor will be Larry Peiperl, who is currently a Clinical Professor at UCSF School of Medicine. He previously worked at *PLOS Medicine* and was the journal's first Senior Research Editor.

*PLOS Medicine* is in rude (translation: excellent) health as I hand it on. This is undoubtedly due to the tremendously talented editors and editorial staff at the journal along with the staff working on other teams within PLOS, as well as our many academic editors, reviewers, and authors. Looking back, I'm gratified to remember how many of these individuals grasped our purpose immediately, supported us enthusiastically, and berated us (equally enthusiastically) when we fell short. I hope they continue to do all three.

Have we succeeded overall? I like to think so.

But this isn't the time to be complacent. Medical publishing, like publishing in general, still has many issues to address, and how these play out over next few years will, I believe, determine the place journals have in the wider dissemination of medical knowledge and, more importantly, its translation into practice and policy.

First, Open Access (OA) to the medical literature has to become universal. By OA I mean, of course, free, immediate access that also allows human and machine reuse without restriction, ensuring that authors are credited. This was the principle on which *PLOS Medicine* was founded; its acceptance, although not without opposition, has gained substantial and sustainable traction. Undoubtedly in some cases there remain practical obstacles to implementation, but the remaining opposition in the industry to the principle of OA to the medical literature is a throwback to the dark days when restricting access was the norm.

Second, the tying of the medical literature to online formats that essentially replicate the limitations of print medium has to change. Journals need to learn from the rest of the Web, where communication has been revolutionized. Articles need to be allowed to evolve, to have new versions, to reflect the dynamic web within which that they now exist. Articles need to be evaluated on their own merit by the most relevant standards. The paper on lethal injection [1] has had just eight academic citations to date; this single measure of impact—of either the article or the journal—misses the paper's demonstrable effect on public policy.

Third, medical journals need to address the inherent conflicts in their way of doing business with the pharmaceutical and associated industries. *PLOS Medicine*'s stance of not taking ads for drugs or devices has allowed us extraordinary freedom. Other journals that continue to rely on funding from these industries cannot lead the debate on what is best for the public's health, such as the imperative to register and report all clinical trials [5] (not just those that companies choose), because their advocacy for the public interest is fettered by their links to proprietary interests

I don't think there's ever been an uninteresting or predictable time in medical publishing. The almost 10 years that I have been at *PLOS Medicine* have been a rollercoaster ride, and my only prediction is that my successor will have an equally exciting time. I'm looking forward to watching the ride.
